# Low Molecular Weight Fucoidan Inhibits Tumor Angiogenesis through Downregulation of HIF-1/VEGF Signaling under Hypoxia 

**DOI:** 10.3390/md13074436

**Published:** 2015-07-17

**Authors:** Meng-Chuan Chen, Wen-Lin Hsu, Pai-An Hwang, Tz-Chong Chou

**Affiliations:** 1 Graduate Institute of Medical Sciences, National Defense Medical Center, Taipei 11483, Taiwan; E-Mail: sport315@gmail.com; 2 School of Medicine, Tzu Chi University, Hualien 97002, Taiwan; E-Mail: hwl@tzuchi.com.tw; 3 Department of Radiation Oncology, Buddhist Tzu Chi General Hospital, Hualien 97002, Taiwan; 4 Seafood Technology Division, Fisheries Research Institute, Council of Agriculture, Keelung 20246, Taiwan; E-Mail: pahwang@mail.tfrin.gov.tw; 5 Institute of Medical Sciences, Tzu Chi University, Hualien 97002, Taiwan; 6 Department of Biotechnology, Asia University, Taichung 41354, Taiwan; 7 China Medical University Hospital, China Medical University, Taichung 40447, Taiwan

**Keywords:** low molecular weight fucoidan, angiogenesis, hypoxia-inducible factor 1 alpha, vascular endothelial growth factor, bladder cancer

## Abstract

Activation of hypoxia-induced hypoxia-inducible factors-1 (HIF-1) plays a critical role in promoting tumor angiogenesis, growth and metastasis. Low molecular weight fucoidan (LMWF) is prepared from brown algae, and exhibits anticancer activity. However, whether LMWF attenuates hypoxia-induced angiogenesis in bladder cancer cells and the molecular mechanisms involved remain unclear. This is the first study to demonstrate that LMWF can inhibit hypoxia-stimulated H_2_O_2_ formation, HIF-1 accumulation and transcriptional activity vascular endothelial growth factor (VEGF) secretion, and the migration and invasion in hypoxic human bladder cancer cells (T24) cells. LMWF also downregulated hypoxia-activated phosphorylation of PI3K/AKT/mTOR/p70S6K/4EBP-1 signaling in T24 cells. Blocking PI3K/AKT or mTOR activity strongly diminished hypoxia-induced HIF-1α expression and VEGF secretion in T24 cells, supporting the involvement of PI3K/AKT/mTOR in the induction of HIF-1α and VEGF. Additionally, LMWF significantly attenuated angiogenesis *in vitro* and *in vivo* evidenced by reduction of tube formation of hypoxic human umbilical vascular endothelial cells and blood capillary generation in the tumor. Similarly, administration of LMWF also inhibited the HIF-1α and VEGF expression *in vivo*, accompanied by a reduction of tumor growth. In summary, under hypoxia conditions, the antiangiogenic activity of LMWF in bladder cancer may be associated with suppressing HIF-1/VEGF-regulated signaling pathway.

## 1. Introduction

Angiogenesis is a physiological process through which new blood vessels grow form existing vessels and is responsible for embryonic development, and tissue organ regeneration. However, uncontrolled and persistent angiogenesis is considered to be closely related to several pathological conditions such as tumor progression. It has been demonstrated that the growth and spread of cancer are highly dependent on angiogenesis for feeding growing tumors with nutrients and oxygen [1,2,3]. Therefore, suppressing tumor angiogenesis is a critical target for preventing or slowing cancer growth.

The presence of a hypoxic area (0.05%–5% O_2_) in a tumor due to its rapid growth is a characteristic feature of most advanced solid tumors. The hypoxic environment in a tumor is a key factor causing the progression and the poor prognosis of cancer because of the aggressive and metastatic cancer phenotypes and therapeutic resistance [4,5]. Notably, hypoxia is a crucial stimulating factor for tumor angiogenesis and metastasis because it increases hypoxia-inducible factors-1 (HIF-1), a nuclear transcription factor, protein accumulation and transcriptional activity [6,7,8]. HIF-1 is a heterodimeric factor consisting of an inducible oxygen-sensitive alpha subunit (HIF-1α) and a constitutive oxygen-insensitive beta subunit (HIF-1β/ARNT). The biological functions of HIF-1 are primarily controlled by the stability of HIF-1, which is tightly regulated by oxygen tension [9]. In normoxic condition, the proline residues of the oxygen-dependent degradation domain (ODDD) in HIF-1α are hydroxylated by prolyl hydroxylase (PHD). The hydroxylated HIF-1α is required for the recognition by the Von Hippel-lindau (VHL) tumor suppressor protein, leading to HIF-1α degradation via ubiquitin-proteasome system (UPS). In contrast, hypoxia greatly decreases the prolyl hydroxylation of HIF-1α resulting from inhibition of PHD activity, thereby increasing the stability and the nuclear level of HIF-1α, where it forms an active complex with HIF-1β and triggers the transcription of pro-angiogenic genes, such as vascular endothelial growth factor (VEGF). The overexpression of HIF-1α has been confirmed in human tumors as compared with that in the respective normal tissues [10], and blocking HIF-1α activation significantly attenuates tumor growth, angiogenesis and progression [8,11,12]. Therefore, reagents with suppressing HIF-1α accumulation and transcriptional activity and/or angiogenesis-related signaling may have a potential to exert an antiangiogenic activity. 

**Figure 1:**
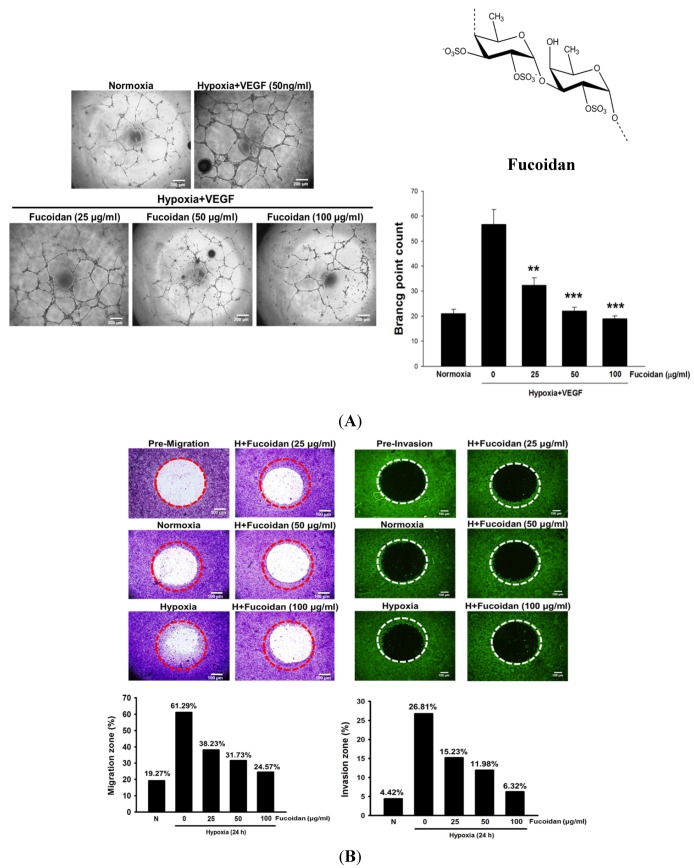
LMWF inhibited the tube formation in HUVECs and the migration and invasion of T24 cells. (**A**) The capillary-like tube formation in HUVECs (**A**) and the migration and invasion of T24 cells (**B**) were determined as described in the Methods section. Data was expressed as mean ± SEM (*n* = 5). ** *P* < 0.01, *** *P* < 0.001 *versus* hypoxia + VEGF-treated alone group.

Brown algae is rich in fucoidan (Figure 1A), a fucose-containing sulfated polysaccharide. Fucoidan exhibits many beneficial functions, including anti-inflammatory, antioxidant, and immunomodulatory activities [13], and is a widely used dietary supplement or nutraceutical. Recently, the anticancer activity of fucoidan associated with induction of apoptosis and modulating the immunity in cancer cells [13,14] has attracted considerable attention. It is noteworthy that the actions of fucoidan are dependent on algal species, the molecular weight and the degree of sulfation. Generally, low molecular weight fucoidan (LMWF) has a greater anticancer activity than high molecular weight fucoidan [15]. In addition, fucoidan could inhibit VEGF-A expression and the binding of VEGF165 to VEGF receptors (VEGFRs) [16,17]. However, whether LMWF attenuates hypoxia-induced tumor angiogenesis and the involvement of HIF-1α remains unknown. This is the first study to demonstrate that the angiogenic activity of LMWF in bladder cancer may be attributed to suppressing the HIF-1α/VEGF signaling pathway. 

## 2. Results 

### 2.1. LMWF Inhibits Hypoxia-Induced Angiogenesis in Human Umbilical Vascular Endothelial Cells (HUVECs) and the Migration and Invasion of Human Bladder Cancer Cells (T24 Cells) 

LMWF dose-dependently reduced hypoxia and VEGF-induced capillary tube-like structure formation in HUVECs (Figure 1A), and did not affect angiogenesis under normoxic conditions (data not shown), suggesting the antiangiogenic activity of LMWF is hypoxia specific. As angiogenesis is essential for tumor metastasis, the effects of LMWF on the migration and invasion of T24 cells were evaluated. As shown in Figure 1B, LMWF treatment greatly inhibited the migration and invasion of hypoxic T24 cells.

### 2.2. LMWF Inhibits Angiogenesis in Vivo and Tumor Growth 

**Figure 2:**
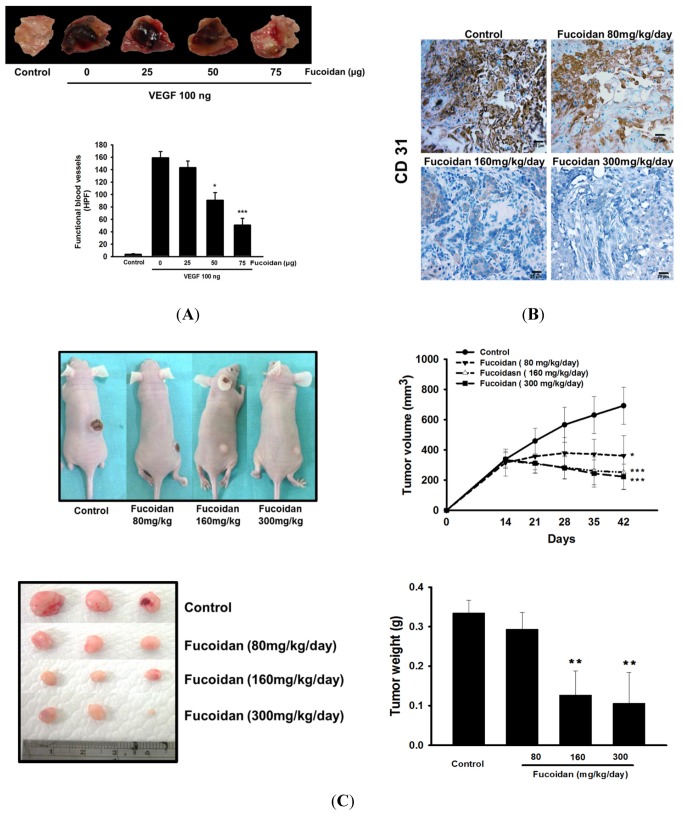
LMWF inhibited tumor angiogenesis and growth *in vivo*. The functional vasculature formation stimulated by VEGF (100 ng) in Matrigel plugs was photographed (200×) (**A**). The CD31-stained capillaries in tumor tissues were identified by immunohistochemistry (**B**). * *P* < 0.05, *** *P* < 0.001 *versus* VEGF-treated alone group. After BALB/c nude mice were injected with T24 cells (s.c.) for 15 days followed by treatment with different doses of LMWF for 30 days, the images of tumor sections, tumor size, and weight were measured (**C**). Data was expressed as mean ± SEM (*n* = 5). * *P* < 0.05, ** *P* < 0.01, *** *P* < 0.001 *versus* untreated cancer mice (Control group).

In the Matrigel containing VEGF, there was remarkable functional vasculature reflected by a much darker color due to being filled with a high amount of intact erythrocytes compared with that of untreated cancer mice (control group) (Figure 2A). Consistently, the amounts of CD31, a specific marker for endothelial cells [18], in tumor tissues were markedly increased (Figure 2B). Administration of LMWF dose-dependently attenuated the angiogenesis *in vivo* as evidenced by decreased vessels in the Matrigel plug and CD31-stained capillaries in the tumor. Moreover, the tumor size and weight were significantly reduced in LMWF-treated mice compared with that in control group (Figure 2C). Additionally, LWMF treatment did not cause body weight loss in mice (data not shown), indicating the safety of LMWF at the dose used.

### 2.3. LMWF Attenuates Hypoxia-Induced HIF-1α Activation, Reactive Oxygen Species (ROS) Formation and VEGF Release in T24 Cells

**Figure 3:**
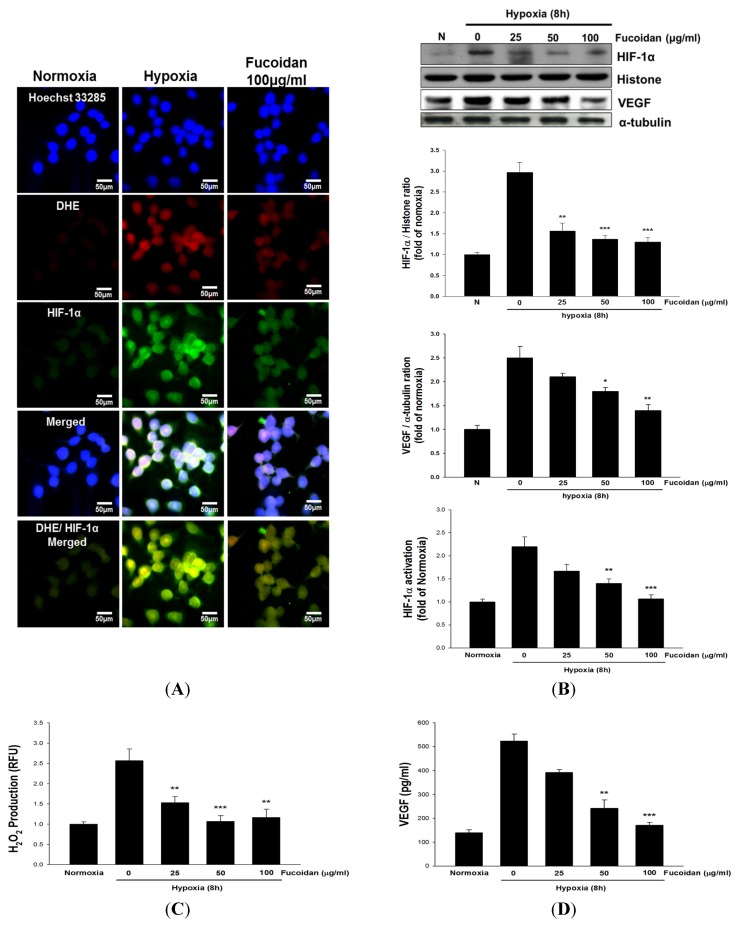
LMWF inhibited hypoxia-induced HIF-1α expression, activation, VEGF and H_2_O_2_ formation in T24 cells. The HIF-1α nuclear translocation (**A**), nuclear HIF-1α protein level (**B**), HIF-1α transcriptional activity (**C**), H_2_O_2_ formation (**D**), VEGF protein expression (**B**) and VEGF release (**E**) of different groups were determined. Data was expressed as mean ± SEM (*n* = 5). * *P* < 0.05, ** *P* < 0.01, *** *P* < 0.001 *versus* hypoxia-treated alone T24 cells.

Exposure of T24 cells to hypoxia for 8 h markedly increased the nuclear translocation of HIF-1α determined by immunofluorescence (Figure 3A) and Western blotting assays (Figure 3B), HIF-1 transcriptional activity (Figure 3C), hydrogen peroxide (H_2_O_2_) formation (Figure 3D), and VEGF expression and release (Figure 3B,E) in T24 cells as compared with that in normoxic T24 cells. However, the alterations caused by hypoxia were dramatically reduced by LMWF.

### 2.4. LMWF Inhibits HIF-1-Regulated Signaling Pathways in T24 Cells

**Figure 4:**
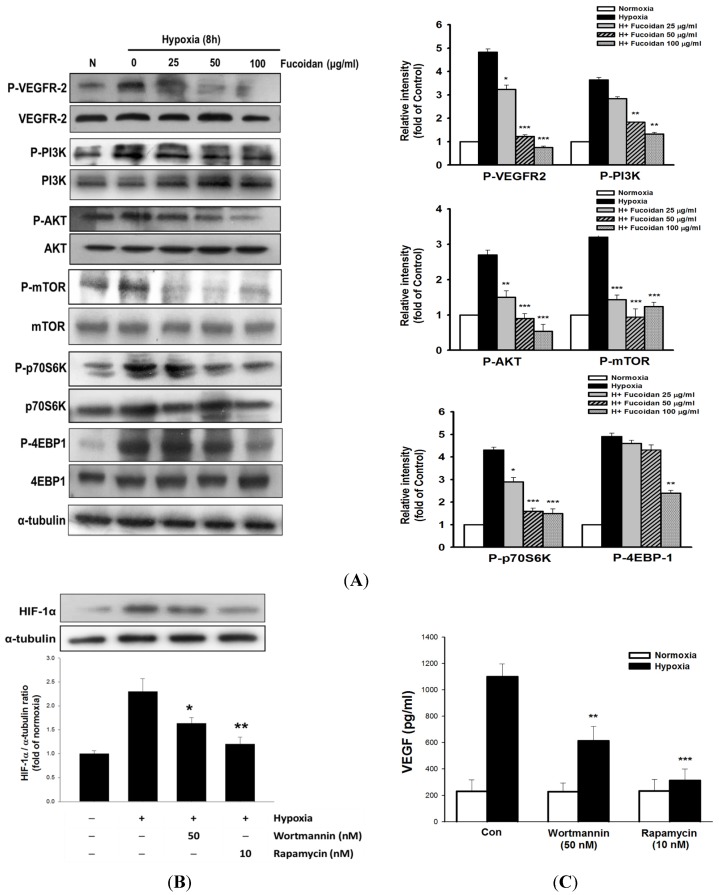
LMWF inhibited VEGF-mediated downstream signaling pathway in T24 cells. The total and phosphorylated target genes were analyzed by Western blotting (**A**). T24 cells were pretreated with rapamycin (10 nM) or wortmannin (50 nM) for 1 h, followed by exposed to normoxia or hypoxia for 8 h, and the alterations of HIF-1 protein expression (**B**), and VEGF secretion (**C**) were determined. Data was expressed as mean ± SEM (*n* = 5). * *P* < 0.05, ** *P* < 0.01, *** *P* < 0.001 *versus* hypoxia-treated alone group.

The hypoxia-induced VEGFR2 activation reflected by increased phosphorylation of VEGFR2 (Figure 4A) in T24 cells was strongly inhibited by LMWF. Subsequently, VEGFR2-activated PI3K/AKT/mTOR signaling pathway can in turn promote HIF-1 protein synthesis through phosphorylation of protein translational regulators, such as phosphorylate ribosomal p70S6kinase (p70S6K1) and 4EBP-1 [19]. As expected, the elevated phosphorylation of AKT, mTOR, p70S6K and 4EBP-1 kinases in T24 cells exposed to hypoxia was significantly diminished by LMWF without affecting the total protein levels of these kinases (Figure 4A). To further examine the role of PI3K/AKT/mTOR signaling in the induction of HIF-1α and VEGF, rapamycin, an inhibitor of mTOR, or wortmannin, an inhibitor of PI3K/AKT, was added. Our data revealed that rapamycin and wortmannin inhibited hypoxia-induced HIF-1α expression and VEGF secretion (Figure 4B,C), indicating the role of this pathway in the regulation of HIF-1α and VEGF transcription. 

### 2.5. LMWF Reduces the Expression of HIF-1α and VEGF in Tumors.

**Figure 5:**
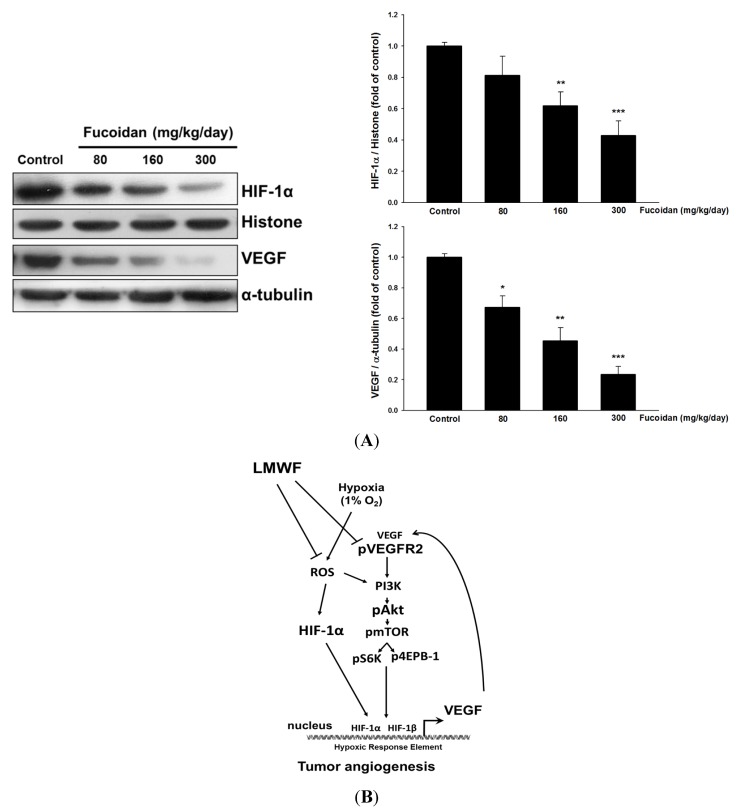
LMWF inhibited nuclear HIF-1α and VEGF protein expression in tumor tissues (**A**). Data was expressed as mean ± SEM (*n* = 5). * *P* < 0.05, ** *P* < 0.01, *** *P* < 0.001 *versus* untreated cancer mice (Control group). The proposed signaling pathways underlying LMWF-mediated antiangiogenesis (**B**). LMWF inhibits hypoxia-induced ROS formation, HIF-1α expression, VEGF secretion, and downstream VEGFR2/PI3K/AKT/mTOR/p70S6K1/4EBP-1 signaling in T24 cells, ultimately suppressing HIF-1α/VEGF transcription and angiogenesis.

Similarly, the increased nuclear HIF-1 protein level and VEGF expression occurring in tumor tissues were significantly attenuated by LMWF treatment compared with that in untreated cancer mice (Figure 5A), suggesting that the antiangiogenic and anticancer activities of LMWF may be related to downregulation of HIF-1 and VEGF. 

## 3. Discussion

The hypoxic tumor environment has been regarded as an important factor leading to genetic and epigenetic adaptation of tumor cells, ultimately promoting tumor progression. HIF-1 is a key mediator of cellular responses to hypoxia, and plays a critical role in enhancing tumor growth by initiating angiogenesis [20,21]. Therefore, targeting hypoxia/HIF-1-driven tumor angiogenesis is a potential strategy for cancer therapy. In the present study, we demonstrated that LMWF treatment greatly reduces angiogenesis both in hypoxic HUVECs and tumor tissues. Furthermore, LMWF is capable of attenuating the nuclear protein accumulation of HIF-1 in hypoxic T24, tumor, and HUVEC cells (data not shown). Thus, the antiangiogenic activity of LMWF may be associated with suppressing HIF-1-mediated processes in tumor cells and surrounding endothelial cells located in hypoxic conditions occurring in most solid tumors. 

The stability of HIF-1 is mainly regulated by PHD, which is responsible for the hydroxylation of specific HIF-1 proline residues and degradation. Notably, O_2_, 2-oxoglutarate and Fe^2+^ are required for PHD full activity [22]. Several lines of evidence have indicated that hypoxia-induced ROS formation, in particular H_2_O_2_, can strongly inhibit the PHD enzymatic activity via a Fenton reaction resulting in an increased proportion of Fe^3+^ in PHD, thereby decreasing PHD activity and increasing HIF-1α accumulation [23]. Like PHD, the activity of HIF asparaginyl hydroxylase known as factor-inhibiting HIF (FIH) is O_2_, 2-oxoglutarate, and Fe^2+^-dependent. Importantly, FIH is a key enzyme accounting for the inhibition of HIF-1α transcriptional activity by hydroxylating the specific asparagine residue (N803) of HIF-1α, subsequently hindering the interaction of HIF-1α with the transcriptional co-activator p300/CBP [24]. Hence, maintaining Fe^2+^ availability through suppression of hypoxia-induced ROS formation is crucial for promoting HIF-1 degradation and diminishing HIF-1 activity by preventing PHD and FIH inactivation. Accordingly, inhibition of H_2_O_2_ formation by LMWF may be a mechanism contributing to the decreased protein levels and activity of HIF-1 in hypoxic T24 cells and tumor.

In the process of HIF-1-induced tumor angiogenesis, the induction and secretion of VEGF is considered a central mediator. Clinical observation has indicated that the expression of VEGF in cancers is highly correlated with tumor progression and poor prognosis [25]. The angiogenic effect of VEGF is mainly mediated by binding and activating (phosphorylation) its receptors especially VEGFR2 [26]. We found that increased expression and secretion of VEGF as well as phosphorylated VEGFR2 were observed in hypoxic T24 cells, which was dramatically inhibited by LMWF, suggesting that LMWF has an ability to block VEGFR2 activation. Upon VEGFR2 activation, the downstream PI3K/AKT/mammalian target of rapamycin (mTOR) signaling pathway is activated. This in turn enhances HIF-1α protein synthesis and VEGF secretion by upregulating protein translational regulators such as p70S6K1 and 4EBP-1 [27,28]. Thus, the VEGFR2 pathway may act as a positive regulating loop to further promote angiogenesis. Treatment with LMWF significantly inhibited the phosphorylation of the AKT/mTOR/p70S6K/4EBP-1 cascade without affecting the total protein levels of these target genes in hypoxic T24 cells, indicating that LMWF specifically inhibited the protein kinase activity. Blocking mTOR or PI3K/AKT activity markedly reversed hypoxia-induced HIF-1α expression and VEGF secretion, strongly supporting the notion that the induction of HIF-1α and VEGF is largely regulated by the PI3K/AKT/mTOR pathway. 

Recent study has indicated that ROS-stimulated HIF-1α accumulation is related to PI3K/AKT activation in hypoxic MCF-7 breast cancer cells [29]. Therefore, in addition to acting PHD, ROS also activates AKT-induced angiogenesis. Together with these findings, the mechanisms accounting for the antiangiogenic effect of LMWF may target different levels, including inhibition of ROS formation, HIF-1α protein accumulation and transcriptional activity, as well as HIF-1α-regulated downstream signaling. Moreover, the nuclear translocation of HIF-1 can be diminished by proteosome inhibitors [30]. Our unpublished data [31] showed that LMWF could inhibit the proteasome activity in cancer mice, which may be another mechanism elevating the HIF-1α protein level. In summary, the present study provides a new molecular mechanism by which LMWF inhibits the angiogenesis, and cancer growth in bladder cancer may be associated with suppressing hypoxia-activated HIF-1 VEGF/VEGFR2-mediated signaling (Figure 5B).

## 4. Materials and Methods 

### 4.1. Chemicals and LMWF Preparation

The antibodies including anti-HIF-1α, anti-VEGF, anti-CD31 (PECAM-1), anti-β-actin, and fluorescein isothiocyanate (FITC)-coupled secondary antibody were purchased from Santa Cruz Biotechnology (Santa Cruz, CA, USA). The anti-VEGFR2, anti-phospho-VEGFR2, anti-AKT, anti-phospho-AKT, anti-mTOR, anti-phospho-mTOR, anti-p70S6K, anti-phospho-p70S6K, anti-4EBP-1 and anti-phospho-4E-BP-1 were purchased from Cell Signaling Technology (Danvers, MA, USA). Horseradish peroxidase (HRP)-labeled secondary antibody and goat anti-rabbit IgG-biotin secondary antibody were obtained from Abcam (Cambridge, MA, USA). Other chemical reagents used in this study were analytical grade and obtained from Sigma (Saint Louis, MO, USA). The present study was approved by Animal Care and Use Committee, National Defense Medical Center, Taipei, Taiwan. The LMWF was a gift from Hi-Q Marine Biotech International Ltd (Xinbei, Taiwan). To prepare the LMWF, fresh dried *Sargassum hemiphyllum* (100 g) was added in 5 L distilled water and boiled at 100 °C for 30 min. Then, the hot water extract was centrifuged and lyophilized under the reduced pressure followed by addition of 4 volumes of 95% ethanol for overnight at 4 °C. For hydrolysis of fucoidan, 5 g of fucoidan was suspended in 125 mL distilled water at 55 °C with at 700 rpm stirring speed followed by addition of glycolytic enzyme at a concentration of 1 mg/g fucoidan for 6 h. After centrifugation at 10,000× *g* for 20 min at 4 °C, the supernatants were passed through a 30 kDa molecular weight cut-off membrane **(**ProStream™ PP, TangenX Technology Co., Boston, MA, USA**)** and the filtrate was further passed through a 1 kDa molecular weight cut-off membrane. Then, the sample was injected into a high-performance size exclusion chromatograph using an Ultrahydrogel 500 column (7.8 × 300 mm, Waters, Milford, MA, USA) to determine the molecular weight distribution [32]. The molecular weight of the final LMWF was mainly 760 Da. The sulfate and fucose content of LMWF was 40.8% ± 0.4% (w/w) and 207.9 ± 0.7 μmol/g, respectively, and the LMWF was dissolved in distilled H_2_O for subsequent tests.

### 4.2. Cell Culture and Hypoxic Treatment

The T24 and HUVECs were purchased from the Bioresource Collection and Research Center (Taipei, Taiwan). T24 cells were incubated in RPMI1640 supplemented with 10% fetal bovine serum (Thermo Fisher Scientific Inc., Waltham, UT, USA), 2 mM l-glutamine, and 100 U/mL penicillin-streptomycin (Gibco, Carlsbad, NM, USA). HUVECs were grown in M199 containing 10% FBS, endothelial cell growth supplement (ECGS, 0.03 mg/mL) and kanamycin (50 U/mL) purchased from Sigma (Saint Louis, MO, USA). For hypoxic exposure, cells were incubated in serum starved medium for 24 h, followed by placing in a sealed hypoxic chamber flushed with a gas mixture of 94% N_2_, 5% CO_2_ and 1% O_2_. 

### 4.3. Capillary-Like Tube Formation Assay

Matrigel (12.5 mg/mL, BD Biosciences, Bedford, MA, USA) was thawed at 4 °C for overnight, and 50 μL Matrigel was quickly pipetted onto 96-well plate and allowed to solidify for 10 min followed by addition of HUVECs (1 × 10^4^ cells/well). After adhesion of the cells, the medium was removed and replaced by fresh medium supplemented with VEGF (50 ng/mL) and various concentrations of LMWF and incubated for 18 h under hypoxic condition. The tube formation was photographed with an Olympus IX 70 invert microscope (Olympus America, Inc., Melville, NY, USA).

### 4.4. Cell Migration and Invasion Assay

The cancer cell migration and invasion were evaluated by using OrisTM 96-well cell migration and invasion assay kits (Platypus Technologies, Madison, WI, USA). Briefly, T24 cells (5 × 10^4^) were seeded onto a 96-well plate sealed with Oris Cell seeding stoppers. After incubation for 12 h, the stoppers were removed and the seeded plates were placed in a hypoxic chamber for 24 h to allow cell migration and invasion into the detection zone. Then, cells were fixed and stained with Wright-Giemsa for migration assay or Calcein AM for invasion assay. Finally, the images were captured using bright field microscopy and then processed by Image J software (version 1.421) (NIH, Bethesda, MD, USA) to determine the area covered by cells in the migration/invasion zone.

### 4.5. Matrigel Plug Angiogenesis Assay

Matrigel (0.5 mL/plug) containing 100 ng VEGF and 20 units heparin with or without of LMWF (25–75 μg) in a liquid form at 4 °C was injected in the midventral abdominal region of 5–6 week-old C57BL/6 mice for 7 days. The intact Matrigel plugs were removed and stained by hematoxylin and eosin (H&E) to identify the formation and infiltration of new microvessels. The number of functional microvessels filled with erythrocytes was counted manually using a microscope in high power field (HPF; 200×).

### 4.6. Xenograft Mouse Model

The 7-week-old female athymic nude mice (BALB/c) weighing ~25 g were used for tumor growth assay. The animal care and experimental procedures were conducted in accordance with the Guiding Principle in the Care and Use of Animals and approved by the Institutional Animal Care and Use Committee of National Defense Medical Center (IACUC 12156). After subcutaneous (s.c) injection of T24 cells (2 × 10^6^ cells per mouse) for 15 days and the tumors reached a palpable size, the mice were administered vehicle (distilled H_2_O) or LMWF (80–300 mg/kg/day, p.o.) for 30 days. The body weight and tumor weight as well as tumor size determined by caliper, following the formula of V = lw^2^/2, wherein l is the length (mm) and w is the width (mm) diameter of tumor were measured. 

### 4.7. Immunohistochemical Staining

Tumor tissues were fixed with 10% formaldehyde and embedded in paraffin followed by incubation of target primary antibody for overnight and goat anti-rabbit IgG-biotin secondary antibody (1:300, Abcam, MA, USA) for 1 h. After extensive washings with PBS, the samples were stained with diaminobenzidine peroxidase substrate and photographed and quantitated using Aperio^®^ software (Leica Biosystems, Vista, CA, USA). 

### 4.8. Immunofluorescence Assay

Cells attached on 8-well plates were treated with the indicated drugs for 8 h under normoxic or hypoxic condition followed by fixation with methanol for 5 min. The non-specific binding sites were blocked with 4% BSA in PBS for 30 min. The cells were incubated with a mouse monoclonal anti-HIF-1α antibody (1:100 in 1% BSA in PBS) for overnight at 4 °C followed by addition of fluorescein isothiocyanate (FITC)-coupled secondary antibody (1:200 in 1% BSA in PBS). The nuclei of cells were stained with Hoechst 33258 dye. After extensive washings with PBS, the coverslips were mounted onto the glass slides and photographed with a fluorescence microscope (Leica, Welzar, Germany).

### 4.9. H_2_O_2_ and Superoxide Measurement

The amount of intracellular H_2_O_2_ was measured by the change of fluorescence resulting from oxidation of 2′,7′-dichlorofluorescein diacetate (H_2_DCF-DA) (Invitrogen Molecular Probes, Carlsbad, CA, USA). Moreover, the dihydroethidium (DHE; 10 μM) was used to detect the superoxide formation and photographed with a fluorescence microscope. 

### 4.10. HIF-1α Activity and VEGF Assay

The HIF-1α activity assay is performed using an HIF-1α Combo Transcription Factor Assay Kit followed the manufacturer's instructions (Cayman, Ann Arbor, MI, USA). The levels of VEGF were determined via a VEGF enzyme-linked immunosorbent assay (ELISA) kit (R&D Systems, Minneapolis, MN, USA).

### 4.11. Western Blotting

The nuclear extracts, and cytosolic extracts were prepared by using NE-PER nuclear and cytoplasmic extraction reagents (Thermo Fisher Scientific Inc., Waltham, UT, USA). The protein samples (30–100 μg) were separated on a 9% SDS-PAGE, and transferred onto nitrocellulose membranes. After blocking with 5% nonfat dry milk in 5% TBST for 1 h, the membranes were incubated with various appropriately diluted primary antibody of target genes for overnight at 4 °C. After washing with TBST, the membranes were incubated with horseradish peroxidase-conjugated secondary antibody for 1 h and the immunoreactivity was visualized by using enhanced HRP substrate luminol reagent (Milipore, Billerica, MA, USA).

### 4.12. Statistical Analysis

The experimental data were expressed as the mean ± SEM of at least three independent experiments. One-way ANOVA with a *post hoc* Bonferroni test was used for statistical analysis. Results were considered to present a significant difference at a value of *P* < 0.05.

## 5. Conclusions

We provide a novel mechanism underlying the anti-angiogenic activity of LMWF that is largely attributed to inhibition of hypoxia-induced oxygen-free radical generation, HIF-1α expression/activity, VEGF secretion, and downstream angiogenesis-related signaling pathways (Figure 5B)
